# Evaluation of 1-Ethyl-3-(3-Dimethylaminopropyl) Carbodiimide Cross-Linked Collagen Membranes for Guided Bone Regeneration in Beagle Dogs

**DOI:** 10.3390/ma13204599

**Published:** 2020-10-15

**Authors:** Jong-Ju Ahn, Hyung-Joon Kim, Eun-Bin Bae, Won-Tak Cho, YunJeong Choi, Su-Hyun Hwang, Chang-Mo Jeong, Jung-Bo Huh

**Affiliations:** 1 Department of Prosthodontics, Dental Research Institute, Dental and Life Science Institute, BK21 PLUS Project, School of Dentistry, Pusan National University, Yangsan 50612, Korea; tarov0414@daum.net (J.-J.A.); 0228dmqls@hanmail.net (E.-B.B.); joonetak@hanmail.net (W.-T.C.); hsh2942@daum.net (S.-H.H.); cmjeong@pusan.ac.kr (C.-M.J.); 2 Department of Oral Physiology, BK21 PLUS Project, Periodontal Diseases Signaling Network Research Center, and Dental and Life Science Institute, School of Dentistry, Pusan National University, Yangsan 50611, Korea; hjoonkim@pusan.ac.kr (H.-J.K.); celinechoi@pusan.ac.kr (Y.C.)

**Keywords:** 1-ethyl-3-(3-dimethylaminopropyl) carbodiimide, cross-linking, collagen membrane, guided bone regeneration, cytotoxicity

## Abstract

The purpose of this study was to evaluate the bone regeneration efficacy of an 1-ethyl-3-(3-dimethylaminopropyl) carbodiimide (EDC)-cross-linked collagen membrane for guided bone regeneration (GBR). A non-cross-linked collagen membrane (Control group), and an EDC-cross-linked collagen membrane (Test group) were used in this study. In vitro, mechanical, and degradation testing and cell studies were performed. In the animal study, 36 artificial bone defects were formed in the mandibles of six beagles. Implants were inserted at the time of bone grafting, and membranes were assigned randomly. Eight weeks later, animals were sacrificed, micro-computed tomography was performed, and hematoxylin-eosin stained specimens were prepared. Physical properties (tensile strength and enzymatic degradation rate) were better in the Test group than in the Control group. No inflammation or membrane collapse was observed in either group, and bone volumes (%) in defects around implants were similar in the two groups (*p* > 0.05). The results of new bone areas (%) analysis also showed similar values in the two groups (*p* > 0.05). Therefore, it can be concluded that cross-linking the collagen membranes with EDC is the method of enhancing the physical properties (tensile strength and enzymatic degradation) of the collagen membranes without risk of toxicity.

## 1. Introduction

Implant placement with guided bone regeneration (GBR) is performed to provide sufficient bone volume and quality at implant recipient sites [1]. GBR requires the use of barrier membranes to prevent the penetration of epithelial cells into bone defects and to maintain defect spaces until they are filled with mature newly formed tissues [2,3,4,5]. An ideal barrier membrane should meet physical and biochemical requirements, be biocompatible, have adequate tissue integration ability, cell occlusion capacity, dimensional stability, and be easily manipulated [6,7,8].

The nonresorbable membranes first used to guide bone regeneration were made of polytetrafluoroethylene (PTFE), expanded PTFE (ePTFE), or titanium [1,9]. These materials are biocompatible and have enough strength to maintain a stable space [10,11], but these nonresorbable membranes also have high exposure rates of 30–40% because of their stiffnesses [12,13,14]. In addition, additional surgery is required to remove these membranes, which presents potential risks to newly formed tissues [15]. To avoid membrane removal surgery and reduce the risk of infection, resorbable membranes were developed [16,17,18].

Collagen membranes derived from animal species are commonly used as resorbable membranes [19]. Collagen is a major structural protein of extracellular matrix and supports the growth of various tissues, and also has the advantages of inducing little inflammation and of being noncytotoxic [20,21]. However, rapid degradation due to the enzymatic activities of polymorphonuclear leukocytes and macrophages, and weak strength can compromise the abilities of these membranes to protect new bone [22,23,24,25], and thus, membranes with improved physical, chemical, and enzymatic-resisting properties have been devised using various cross-linking techniques [22,26,27,28]. According to Moses et al. [29], cross-linked collagen membranes have slower degradation rates than their non-cross-linked counterparts. Bornstein et al. [30] assessed the bone regenerative abilities of cross-linked and non-cross-linked collagen membranes made of the same type of collagen, by covering porcine calvaria defects after bone grafts, and reported new bone regenerations of 27 ± 15% and 36 ± 9% for non-cross-linked and cross-linked collagen membranes, respectively.

Various cross-linking methods such as dehydrothermal, chemical, and ultraviolet irradiation-based treatments have been introduced [31,32,33]. Of these, cross-linking using chemical agents such as glutaraldehyde (GTA), 4-arm polyethylene glycol succinimidyl glutarate (4SP), diphenyl phosphoryl azide (DPPA), genipin, and 1-ethyl-3-(3-dimethylaminopropyl) carbodiimide (EDC) are most commonly used, because other cross-linking methods do not cross-link collagen well enough to increase mechanical stability and slow down membrane degradation [34,35,36]. Although several studies have reported that chemical cross-linking methods can trigger foreign body response due to chemical agent residues [33,37], EDC tends to lower cytotoxicity, because it does not remain as part of the linkage, and is converted to water-soluble urea derivatives that are relatively noncytotoxic [31,38,39,40]. In a previous study in which two different bone grafts were implanted into rat calvarial defects with EDC-cross-linked collagen membrane, animals showed excellent bone regeneration, no inflammatory reaction, and no membrane collapse [41].

Although several studies have examined the bone-regenerative effects of EDC-cross-linked membranes, most were laboratory or small animal studies [33,37,41]. Therefore, the aim of the present study was to assess the physiochemical properties of EDC-cross-linked collagen membranes and to evaluate their effects on GBR in a canine implant defect model.

## 2. Materials and Methods

### 2.1. Experimental Materials and Their Characterization

#### 2.1.1. Experimental Materials

Two types of resorbable collagen membranes were used in this study: Non-cross-linked collagen membranes (Bio-Gide^®^, Geistlich, Wolhusen, Switzerland) derived from porcine skin, which were used in the Control group; and EDC-cross-linked collagen membranes (Colla-D^®^, MedPark, Busan, Korea) derived from bovine tendon, which were used in the Test group.

#### 2.1.2. Field Emission Scanning Electron Microscopy (FE-SEM) 

Morphologies in the Control and Test groups were determined by FE-SEM (Supra 25, Zeiss, Jena, Germany). Samples from both groups were sputter-coated with Au (SCD 005, BAL-TEC, Balzers, Liechtenstein) and observed at magnifications of ×200, ×500, and ×5000.

#### 2.1.3. Energy-Dispersive X-ray Spectroscopy (EDS) Analysis 

An energy-dispersive X-ray spectrometer (EDS) equipped with a silicon drift detector (X-MaxN 80, Oxford, Cambridge, UK) and an energy microanalysis program (Aztec, Oxford, Cambridge, UK), connected to an FE-SEM, was used to determine membrane elemental distributions.

#### 2.1.4. Tensile Strength Test

A universal testing machine (TO-102, Testone, Korea) was used to determine membrane tensile strengths. Samples (10 mm × 40 mm) were immersed in distilled water for 24 h prior to testing. Ten millimeters of each end of the membranes were pegged, and testing was conducted at an elongation rate of 20 mm/min [42].

#### 2.1.5. Degradation Test

To evaluate membrane degradation, membranes (*n* = 3) were cut to 10 × 10 mm and weighed. The test solution (5 mL), which contained 18 units/mg collagenase, was incubated at 37 °C for 1 h. After 1 h, the membrane samples were put into each solution [39]. After incubating samples for 1, 2, 4, 6, 9, 12, 24, or 48 h at 37 °C, the solution was carefully removed, and samples were freeze-dried for 24 h at −40 °C. The weights of dried samples were measured, and membrane degradations were expressed as percentage (%) weight losses.

### 2.2. In Vitro Study

#### 2.2.1. Cell Cultures

Human gingival fibroblasts (hGnFs) were obtained from ScienCell Research Laboratories (Carlsbad, CA, USA) and cultured in Fibroblast Medium (FM; ScienCell Research Laboratories, Carlsbad, CA, USA) in a humidified 5% CO_2_ atmosphere at 37 °C. Culture medium was renewed every 48 h.

#### 2.2.2. Proliferation Assay

hGcF proliferative activity was assessed using a Cell Counting Kit-8 (CCK-8; Dojindo Laboratories, Kumamoto, Japan). Briefly, cells were seeded at a density of 2000 cells per well in 48-well plates. Control group and Test group membranes were preincubated with 500 μL FM before being seeded with cells. All conditions were evaluated in triplicate. After 24, 48, and 72 h, medium was discarded and replaced with 200 μL FM containing 10% CCK-8 solution, according to the manufacturer’s instructions, and were incubated at 37 °C for 2 h in a humidified 5% CO_2_ atmosphere. Absorbances at 450 nm were measured using a Benchmark microplate reader (Bio-Rad Laboratories, Hercules, CA, USA).

#### 2.2.3. Immunocytochemistry

The adhesion of hGnFs to membranes 2 h after seeding was observed by immunofluorescence imaging. First, hGnFs were seeded at a density of 2000 cells per membrane in 48-well plates. After 2 h, the cells were washed twice with phosphate buffered saline (PBS) and fixed in 3.7% formaldehyde solution in PBS for 20 min at room temperature. Cells were then washed three times with PBS, incubated in PBS containing 0.1% TritonX-100 (Sigma, St. Louis, MO, USA) for 5 min, washed three times with PBS, mounted on slide glasses on membranes, and incubated with 100 nM rhodamine phalloidin (Invitrogen, Corp., Carlsbad, CA, USA) for 30 min. After washing with PBS, cells were incubated with 10 μg/mL 4′,6-diamidino-2-phenylindole (DAPI; Invitrogen, Corp., Carlsbad, CA, USA) for 10 min. Images were acquired using a Nikon Eclipse TE2000-E confocal microscope (Nikon, Tokyo, Japan).

### 2.3. In Vivo Study

#### 2.3.1. Surgical Procedures

Six adult male beagle dogs, over 3 years old and weighing more than 10 kg, were used in this study, which was approved by the Chonnam National University Animal Experimental Ethics Committee (CNU IACUC-YB-2018-94). Mandibular premolars and the 1st and 2nd molars of each dog were extracted bilaterally. All surgical procedures, including extractions, implant surgery, the bone graft procedure, and membrane placement, were performed under general anesthesia using 5 mg/kg Zoletil 50 (Virbac Lab., Carros, France) and 10 μg/kg of medetomidine (Tomidin, Provet, Turkey) by intramuscular injection followed by insufflation narcosis, which was maintained with 2–3% Sevoflurane (Hana Pharm, Seoul, Korea). Additional local anesthesia was performed using 0.005% bupivacaine (Myungmoon Pharm, Seoul, Korea). Pain was controlled by administering 2 mg/kg tramadol (MARITROL, Jeil Pharm, Daegu, Korea) and 2.2 mg/kg carprofen (Rimadyl, Zoetis, Parsippany, NJ, USA) by intravenous injection.

**Figure 1:**
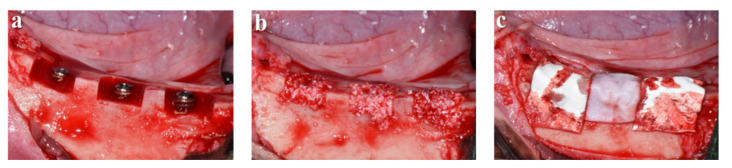
Surgical procedure to perform guided bone regeneration (GBR) in beagle dogs. (**a**) Implants placed in bone defect areas; (**b**) bone grafting; (**c**) membrane placement after bone grafting.

Implant surgery was performed 2 months after extraction. A crestal incision and a vertical releasing incision were made at edentulous sites. Three rectangular bone defects (8 mm wide, 5 mm high, 5 mm deep) were prepared under saline irrigation. A total of 36 implants (diameter: 4 mm, length: 8 mm, Cowell Medi Co, Ltd., Busan, Korea) were inserted with 3 threads exposed (Figure 1a). All peri-implant defective sites were grafted with porcine-derived xenografts (Bone-XP, MedPark, Busan, Korea) (Figure 1b), and then randomly covered with either Control group or Test group membranes (Figure 1c). Surgical sites were sutured with 4-0 Vicryl (Mersilk, Ethicon Co., Livingston, UK). Before surgery, each dog was injected with 20 mg/kg of antibiotic cefazolin sodium (Chong Kun Dang, Seoul, Korea), and after surgery with 10 mg/kg of methylprednisolone succinate sodium (Reyon Pharm, Seoul, Korea). Post-operative treatments were orally administered for 2 weeks, that is, amoxicillin-clavulate (Amocla, Kuhnil Pharm, Chungnam, Korea) as an antibiotic, firocoxib (Previcox, Merial, France) 5 mg/kg as an anti-inflammatory, and 0.5 mg/kg famotidine (Nelson famotidine, Nelsonkorea, Seoul, Korea) for gastrointestinal protection. All dogs were monitored daily, and sutures were removed one week after surgery. Dogs were sacrificed at 8 weeks after surgery, and mandibles were carefully harvested and fixed in formalin (Sigma Aldrich Co., St. Louis, MO, USA) for 2 weeks.

#### 2.3.2. Micro-Computed Tomography (µCT) Analysis

Harvested mandibles were prepared for micro-CT analysis to determine bone volumes in the regions of interest (ROIs), 1 mm around the implant excluding the base of the implant. All specimens were scanned at 130 kV and 60 µA using a micro-CT scanner (Skyscan-1173, ver. 1.6, Bruker-CT Co., Kontich, Belgium) and a pixel size of 24.15 µm. All images were reconstructed using Nrecon reconstruction software ver. 1.7.0.4 (Bruker-CT Co., Kontich, Belgium).

#### 2.3.3. Histological and Histomorphometric Analysis

**Figure 2:**
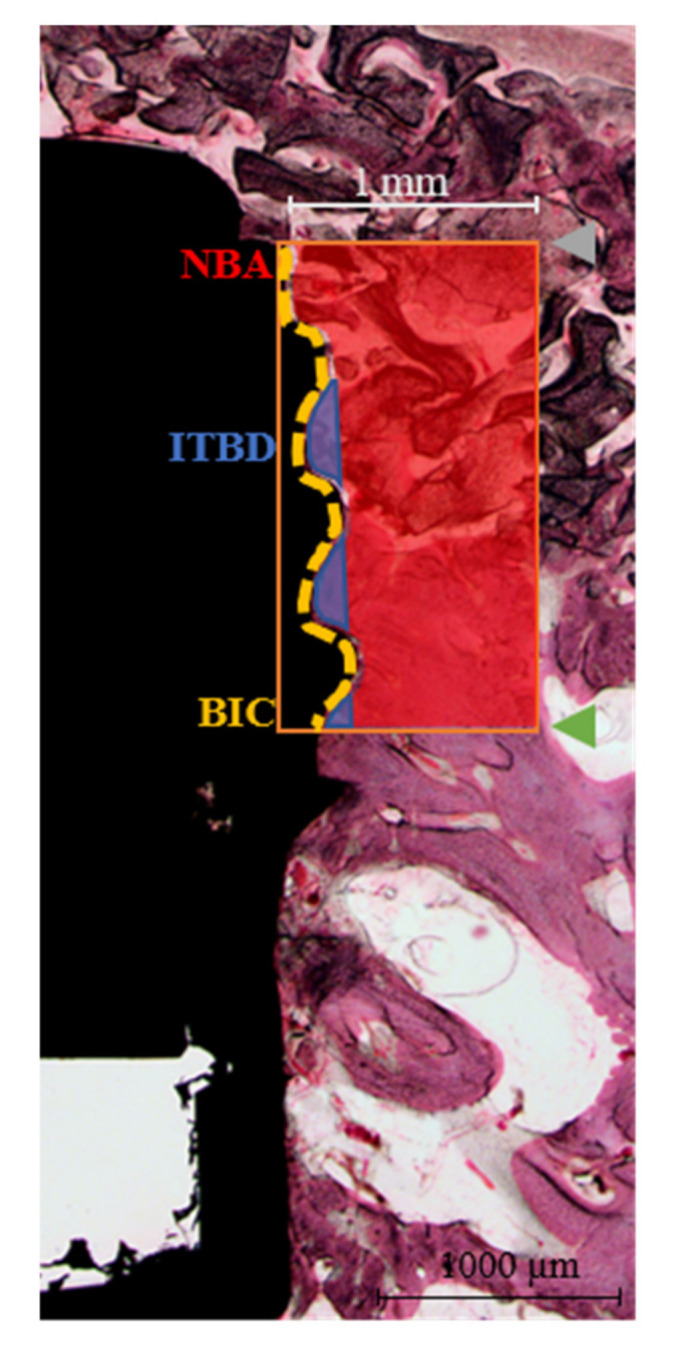
Histomorphometric parameters around the implants for analyzing new bone generation rates. The region of interest (orange rectangle) was set at a width of 1 mm, and height was defined as the distance from the implant platform and to the third thread of the implant fixture. NBA, new bone area; ITBD, inter-thread bone density; BIC, bone-to-implant contact; gray point, implant platform; green point, third thread of implant fixture; red area, NBA; blue area, ITBD; yellow line, BIC line.

After micro-CT analysis, all specimens were dehydrated using ethanol series (70%, 80%, 90%, 100%) and infiltrated with Technovit 7200 resin (Heraeus KULZER, Hanau, Germany) for a week. To make polymerized specimen blocks, specimens were fixed on a frame and then embedded using the UV embedding system (Exakt 520, Kulzer), according to the manufacturer’s instructions. Polymerized blocks were sectioned at implant centers using a diamond cutter (KULZER EXAKT 300 CP Band System, Exakt Apparatebau, Norderstedt, Germany). Sectioned specimens were ground to 30 µm using a grinding machine (KULZER EXAKT 400CS, Exakt Apparatebau, Norderstedt, Germany), mounted on slides, and stained with hematoxylin and eosin (H&E). Images of stained specimens were captured using a computer connected to a light microscope (Olympus BX, Olympus, Tokyo, Japan). Percentage New Bone Areas (NBA%), Inter-Thread Bone Densities (ITBD%), and percentage Bone-Implant Contacts (BIC%) were determined using i-solution (IMT, Daejeon, Korea) by a single investigator (Figure 2).

- NBA (%) = New bone area in the 1 mm-wide rectangle from the top of the implant fixture platform to the third thread (mm^2^)/total area of the 1 mm-wide rectangle (mm^2^) × 100
- ITBD (%) = New bone area between the first to third threads of the implant fixture (mm^2^)/total area between the first to third threads (mm^2^) × 100
- BIC (%) = Length of contact with new bone (mm)/total length from the platform to the third implant thread (mm) × 100

### 2.4. Statistical Analysis

The Mann–Whitney U test was used to analyze in vitro and in vivo results. Results are presented as means ± standard deviations (SD) and the analysis was conducted using SPSS ver. 25.0 (SPSS Inc, Chicago, IL, USA). Statistical significance was accepted for *p* values < 0.05.

## 3. Results

### 3.1. Characterization Results

#### 3.1.1. Morphological Findings

**Figure 3:**
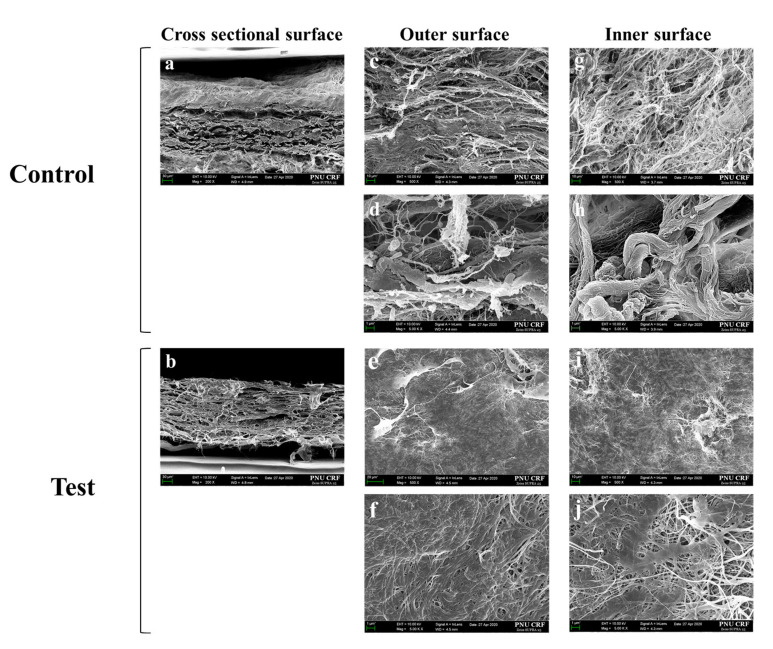
FE-SEM images of both membranes in three directions. (**a**,**c**,**d**,**g**,**h**) Non-cross-linked membrane; (**b**,**e**,**f**,**i**,**j**) cross-linked membrane. (**a**,**b**) Cross-sectional surface images; (**c**,**d**,**e**,**f**) outer surface images; (**g**,**h**,**i**,**j**) inner surface image. Original magnifications: ×200 (**a**,**b**); ×500 (**c**,**e**,**g**,**i**); ×5000 (**d**,**f**,**h**,**j**). Control is represented by non-cross-linked collagen membrane; Test is represented by EDC-cross-linked collagen membrane.

FE-SEM images of cross-sections and surface morphologies of Control group and Test group membranes are shown in Figure 3. Both membrane types showed similarly shaped contacts with outer (contact with soft tissue) and inner surfaces (contact with the hard tissue), respectively, but the surface density of the Test group was higher than that of the Control group (Figure 3c,e,g,i). Both groups were interwoven individual collagen fibers that formed irregular collagen strands (Figure 3d,f,h,j).

#### 3.1.2. EDS Analysis

Chemical composition analyses of both groups revealed similar atomic percentages (Table 1). Control group membranes were composed of carbon (57.67%), oxygen (21.7%), nitrogen (20.51%), and calcium (0.13%). Likewise, Test group membranes were composed of carbon (58.88%), oxygen (21.74%), and nitrogen (19.38%).

#### 3.1.3. Tensile Strength Test

Means ± standard deviations (SD) of tensile strengths of the Control group and Test group membranes were 11.46 ± 1.90 and 16.70 ± 2.43, respectively (Table 2), and these values were significantly different (*p* < 0.05).

#### 3.1.4. Enzymatic Degradation Test

**Figure 4:**
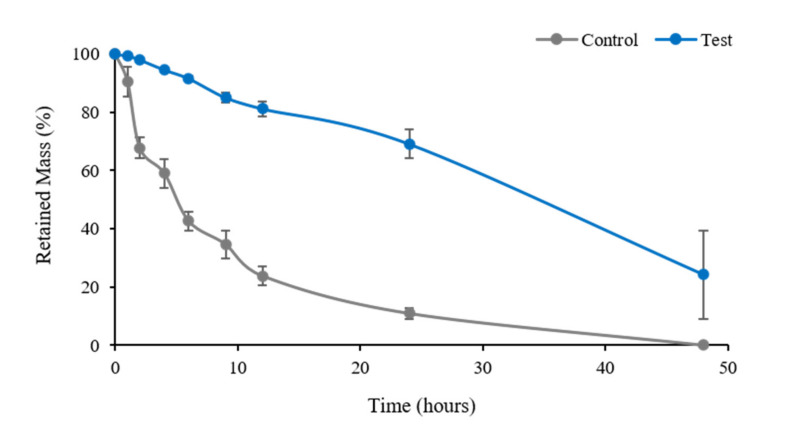
Results of comparative experiment on enzymatic degradation of two types of membranes. Control is represented by non-cross-linked collagen membrane; Test is represented by EDC-cross-linked collagen membrane.

The results of enzyme resistance testing are shown in Figure 4. Control group membranes continuously degraded during testing and disappeared at 48 h, whereas Test group membranes degraded more slowly until 24 h and then degraded more rapidly. At 48 h, Test membranes were partially degraded. These results showed that the Test group membrane was significantly more resistant to enzymes than the Control group membrane (*p* = 0.043).

### 3.2. In Vitro Results

#### 3.2.1. Proliferation Assay

**Figure 5:**
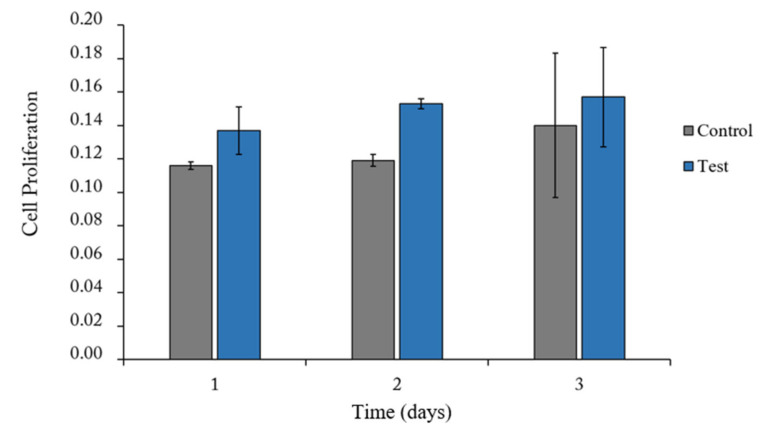
The results of proliferation of hGnF cells over 1, 2, and 3 days of culture using each membrane. Control is represented by non-cross-linked collagen membrane; Test is represented by EDC-cross-linked collagen membrane.

One day after seeding, hGnF cells appeared to have attached to the membrane surfaces. Cell proliferations on Control group and Test group membranes were similar at all times (*p* > 0.05) (Figure 5).

#### 3.2.2. Immunofluorescent Staining

**Figure 6:**
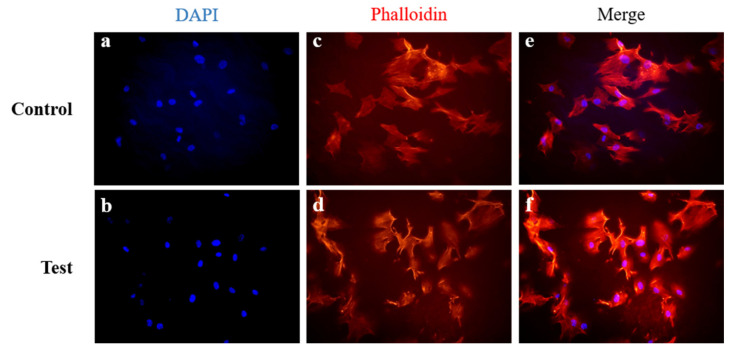
Immunofluorescent staining of human gingival fibroblasts (hGnFs) on membranes 2 h after seeding. (**a**,**b**) DAPI staining of cell nuclei; (**c**,**d**) rhodamine phalloidin staining of F-actin; (**e**,**f**) merged image; (**a**,**c**,**e**) non-cross-linked collagen membrane; (**b**,**d**,**f**) EDC-cross-linked collagen membrane. Control is represented by non-cross-linked collagen membrane; Test is represented by EDC-cross-linked collagen membrane.

Immunocytochemical staining of total actin and DAPI revealed that cells adhered equally to both membranes 2 h after seeding (Figure 6).

### 3.3. In Vivo Results

#### 3.3.1. Clinical Findings

All six experimental animals survived, and no infection or inflammation was observed at surgical sites. The 36 implants were collected without issue.

#### 3.3.2. µCT Findings

Well-formed bone was observed in both groups. Bone volume results are summarized in Table 3. Mean (±SD) bone volumes (%) in the Control and Test groups were similar (62.19 (±9.97) and 60.98 (±10.02), respectively). 

#### 3.3.3. Histological Findings

**Figure 7:**
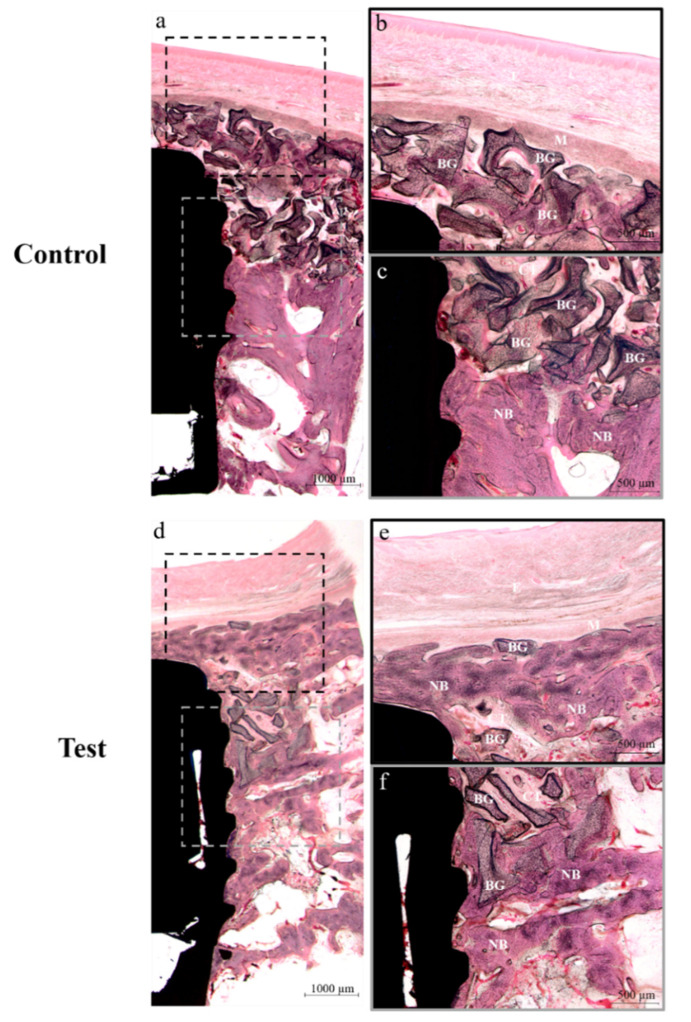
Histological sections images stained with hematoxylin-eosin (H&E) obtained after transplantation for 8 weeks. (**a**–**c**) Control group, (**d**–**f**) Test group. E, Epithelium; M, Membrane; BG, Bone grafts; CT, Connective tissue; NB, New bone. Control is represented by non-cross-linked collagen membrane; Test is represented by EDC-cross-linked collagen membrane. Original magnification: ×12.5 (**a**,**d**); ×40 (**d**,**c**,**e**,**f**).

The histological specimens are shown in Figure 7. In both groups, no adverse reaction was observed (Figure 7a,d). Both membrane types partially survived for 8 weeks in situ (Figure 7b,e), and good healing with new bone formation was observed in both groups (Figure 7c,f).

#### 3.3.4. Histomorphometric Findings

The results of histomorphometric analysis are summarized in Table 4. New bone area (NBA, %) values (24.78 ± 12.15% and 23.46 ± 8.52%; *p* > 0.05), inter-thread bone densities (ITBD, %) (56.98 ± 12.68% and 42.68 ± 21.89%; *p* > 0.05), and bone-to-implant contact percentages (BIC%) (50.44 ± 11.19% and 48.24 ± 13.19%; *p* > 0.05) were similar in the Control and Test groups. 

## 4. Discussion

Ideal membranes can prevent foreign body response and epithelial cell invasion [15], and ideal membranes can remain in situ until enough periodontal tissue and bone have regenerated [43]. Moses et al. [44] reported that cross-linked collagen membranes better assist bone healing by withstanding enzymatic degradation during the bone regeneration period. EDC is one of the most widely used agents to cross-link collagen membranes. Park et al. [39] showed that the EDC-cross-linked membranes have lower cytotoxicity and greater enzymatic degradation resistance than their non-cross-linked counterparts in vitro. Others have demonstrated the biocompatibility of cross-linked collagen membranes [38,45,46].

Appropriate mechanical properties enable membranes to survive in vivo and to supply the stress needed to encourage early tissues to differentiate into pre-osteoblasts [15,31]. In the present study, EDC-cross-linked membranes had greater tensile strengths than non-cross-linked controls (Table 2) (*p* < 0.05), which is consistent with the results of previous studies [39,40,47,48].

Enzymatic degradation resistance is another important contributor to surgical success [49]. During the immediate postoperative period, membrane collapse and degradation can adversely affect bone formation [50]. Therefore, to achieve satisfactory GBR results, membrane degradation rates must be regulated [50]. We found that cross-linked membranes better resisted enzymatic degradation (Figure 4). Non-cross-linked membranes degraded steadily after implantation, whereas cross-linked membranes remained relatively intact at 20 days postoperatively. This result is in line with those of previous studies [40,47,49], and shows that EDC-cross-linking strengthened interactions between collagen molecules and increased structural integrity [50,51]. As our animal experiments were conducted using a short follow-up of 8 weeks, it was difficult to confirm the difference between the in vivo biodegradabilities of the two membranes histologically. We suggest this be further investigated by longer-term study.

The cytotoxicities of chemically cross-linked membranes are determined by the presence of residual chemical species [39], and thus, post-treatments such as washing, and evaporation are used to reduce the risk of cytotoxicity [40]. In our in vitro studies, we used human gingival fibroblasts (hGnFs), which are the main interstitial cells and important for maintaining the original shape and function of gums, and have also been used to evaluate cell proliferation on different membrane types [52]. In the present study, cell viability results confirmed that cross-linked and non-cross-linked membranes performed similarly in terms of cell adhesion and proliferation (Figure 5). In addition, immunocytochemistry showed that hGnF cells adhered equally well to both membranes (Figure 6). Furthermore, our cytotoxicity investigation showed cross-linked and non-cross-linked membranes were both noncytotoxic, and these results concurred with previous studies [53,54]. In addition, through the EDS analysis, it can be confirmed that both membranes (cross-linked and non-cross-linked membranes) have similar chemical compositions (Table 2). This result appears to be that the chemical components of EDC have been removed by washing because EDC does not remain as part of the bond and turns into a water-soluble urea derivative with low cytotoxicity [39,55]. Therefore, as previously reported [39,47], we believe that EDC-cross-linked collagen membranes do not raise cytotoxicity concerns.

The histological results of bone defects around implants showed that both membranes integrated well with surrounding tissues and achieved good osseointegration (Figure 7). In addition, no membrane exposure was observed at any animal, and all defects healed without complications. Histomorphometric and micro-CT analyses showed that cross-linked and non-cross-linked membranes had similar bone regeneration abilities (Table 3 and Table 4). These results are contrary to a previous study in which the cross-linked collagen membrane showed a relatively high bone regeneration rate compared to the non-cross-linked collagen membrane [30]. However, the previous study was conducted with a larger-size defect (6 × 6 × 6 mm and 9 × 9 × 9 mm) than that applied in this study (8 × 5 × 5 mm) [30]. In addition, the results of the previous study have shown that the difference in bone regeneration rates between the cross-linked collagen membrane and the non-cross-linked membrane at the relatively wide defect size (9 × 9 × 9 mm) tended be relatively higher than that of the narrow defect size (6 × 6 × 6 mm) [30]. Therefore, it is considered that the defect size applied in this study was not sufficient for the beneficial effect on bone regeneration of the cross-linked collagen membrane. In addition, the previous study had a healing period of 16 weeks [30]. Thus, it is expected that additional studies with various defect-size models and a long healing period are needed. Notably, the origins of these two collagen membranes differed, that is, Colla-D^®^ (cross-linked) was composed of bovine collagen, and Bio-Gide^®^ (non-cross-linked) was composed of porcine collagen. Bio-Gide^®^ was selected as controls because previous studies have reported good results in terms of biocompatibility and bone regeneration [30,38,56,57], and because it is the most widely used resorbable membrane [11].

## 5. Conclusions

Given the limitations of the present study, we conclude that: (i) EDC-cross-linked collagen membranes are not cytotoxic; (ii) EDC-cross-linked collagen membranes have better mechanical properties and enzymatic degradation resistance than non-cross-linked collagen membranes; and (iii) EDC-cross-linked and non-cross-linked collagen membranes similarly aid bone regeneration. Therefore, the EDC-cross-linking of collagen membranes can be considered a means of improving membrane physical characteristics for guided bone generation without risk of toxicity.

## Figures and Tables

**Table 1:** Atomic percentages of the two membrane types as determined by EDS (%).

Elements	Group	
	Control	Test
C	57.67	58.88
O	21.7	21.74
N	20.51	19.38
Ca	0.13	0

Control is represented by non-cross-linked collagen membrane; Test is represented by EDC-cross-linked collagen membrane.

**Table 2:** Tensile strength of membranes in wet state (unit: N) (*n* = 5).

Group	Mean	SD	*p-*Value
Control	11.46	1.90	0.043
Test	16.70	2.43	

Control is represented by non-cross-linked collagen membrane; Test is represented by EDC-cross-linked collagen membrane.

**Table 3:** Bone volume analysis within regions of interest (ROIs) (%) (*n* = 18).

Groups	Mean	SD	*p*-Value
Control	62.19	9.97	0.815
Test	60.98	10.02	

Values are expressed as percentages. Control is represented by non-cross-linked collagen membrane; Test is represented by EDC-cross-linked collagen membrane.

**Table 4:** Histometric analysis within regions of interest (%) (*n* = 18).

Group	Mean ± SD		
	NBA	ITBD	BIC
Control	24.78 ± 12.15	56.98 ± 12.68	50.44 ± 11.19
Test	23.46 ± 8.52	42.68 ± 21.89	48.24 ± 13.19
*p-*value	0.874	0.179	0.268

Values are expressed as percentages. NBA, new bone area %; ITBD, inter-thread bone density %; BIC%, percentage bone-to-implant contact. Control is represented by non-cross-linked collagen membrane; Test is represented by EDC-cross-linked collagen membrane.

## References

[1] Tal H., Kozlovsky A., Artzi Z., Nemcovsky C.E., Moses O. (2008). Cross-linked and non-cross-linked collagen barrier membranes disintegrate following surgical exposure to the oral environment: A histological study in the cat. Clin. Oral Implant. Res..

[2] Hammerle C.H., Karring T. (1998). Guided bone regeneration at oral implant sites. Periodontol. 2000.

[3] Hammerle C.H.F., Lang N.P. (2001). Single stage surgery combining transmucosal implant placement with guided bone regeneration and bioresorbable materials. Clin. Oral Implant Res..

[4] Saghiri M.A., Asatourian A., Garcia-Godoy F., Sheibani N. (2016). The role of angiogenesis in implant dentistry part II: The effect of bone-grafting and barrier membrane materials on angiogenesis. Med. Oral Patol. Oral Cir. Bucal..

[5] Buser D., Hoffmann B., Bernard J.P., Lussi A., Mettler D., Schenk R.K. (1998). Evaluation of filling materials in membrane—protected bone defects. A comparative histomorphometric study in the mandible of miniature pigs. Clin. Oral Implant. Res..

[6] Dahlin C., Linde A., Gottlow J., Nyman S. (1988). Healing of bone defects by guided tissue regeneration. Plast. Reconstr. Surg..

[7] Hammerle C.H.F., Jung R.E. (2003). Bone augmentation by means of barrier membranes. Periodontol. 2000.

[8] Retzepi M., Donos N. (2010). Guided Bone Regeneration: Biological principle and therapeutic applications. Clin. Oral Implant. Res..

[9] Wessing B., Lettner S., Zechner W. (2018). Guided Bone Regeneration with Collagen Membranes and Particulate Graft Materials: A Systematic Review and Meta-Analysis. Int. J. Oral Maxillofac Implant..

[10] Wiltfang J., Merten H.A., Peters J.H. (1998). Comparative study of guided bone regeneration using absorbable and permanent barrier membranes: A histologic report. Int. J. Oral Maxillofac Implant..

[11] Jiménez García J., Berghezan S., Caramês J.M.M., Dard M., Marques D.J.J. (2017). Effect of cross-linked vs non-cross-linked collagen membranes on bone: A systematic review. J. Periodontal. Res..

[12] Lang N.P., Hammerle C.H., Bragger U., Lehmann B., Nyman S.R. (1994). Guided tissue regeneration in jawbone defects prior to implant placement. Clin. Oral Implant. Res..

[13] Carpio L., Loza J., Lynch S., Genco R. (2000). Guided bone regeneration around endosseous implants with anorganic bovine bone mineral. A randomized controlled trial comparing bioabsorbable versus non-resorbable barriers. J. Periodontol..

[14] Becker W., Dahlin C., Becker B.E., Lekholm U., van Steenberghe D., Higuchi K., Kultje C. (1994). The use of e-PTFE barrier membranes for bone promotion around titanium implants placed into extraction sockets: A prospective multicenter study. Int. J. Oral Maxillofac Implant..

[15] Hardwick R., Hayes B.K., Flynn C. (1995). Devices for dentoalveolar regeneration: An up-to-date literature review. J. Periodontol..

[16] Rowe M.J., Kamocki K., Pankajakshan D., Li D., Bruzzaniti A., Thomas V., Blanchard S.B., Bottino M.C. (2016). Dimensionally stable and bioactive membrane for guided bone regeneration: An in vitro study. J. Biomed. Mater. Res. B Appl. Biomater..

[17] Zitzmann N.U., Naef R., Scharer P. (1997). Resorbable versus nonresorbable membranes in combination with Bio-Oss for guided bone regeneration. Int. J. Oral Maxillofac Implant..

[18] McAllister B.S., Haghighat K. (2007). Bone augmentation techniques. J. Periodontol..

[19] Aldemir Dikici B., Dikici S., Reilly G.C., MacNeil S., Claeyssens F.A. (2019). Novel Bilayer Polycaprolactone Membrane for Guided Bone Regeneration: Combining Electrospinning and Emulsion Templating. Materials.

[20] Park J.H., Park C.K., Kim E.S., Park S.Y., Jo C.M., Tak W.Y., Kweon Y.O., Kim S.K., Choi Y.W. (2003). The diagnostic value of serum hyaluronic acid, 7S domain of type IV collagen and AST/ALT ratio as markers of hepatic fibrosis in chronic hepatitis B and cirrhosis patients. Korean J. Hepatol..

[21] Gentile P., Chiono V., Tonda-Turo C., Ferreira A.M., Ciardelli G. (2011). Polymeric membranes for guided bone regeneration. Biotechnol. J..

[22] Brunel G., Piantoni P., Elharar F., Benqué E., Marin P., Zahedi S. (1996). Regeneration of rat calvarial defects using a bioabsorbable membrane technique: Influence of collagen cross-linking. J. Periodontol..

[23] Schwarz F., Rothamel D., Herten M., Sager M., Becker J. (2006). Angiogenesis pattern of native and cross-linked collagen membranes: An immunohistochemical study in the rat. Clin. Oral Implant. Res..

[24] Lee C.H., Singla A., Lee Y. (2001). Biomedical applications of collagen. Int. J. Pharm..

[25] Peinemann K.-V., Nunes S.P. (2011). Membrane Technology. Membranes for Life Sciences.

[26] Zahedi S., Legrand R., Brunel G., Albert A., Dewe W., Coumans B., Bernard J.P. (1998). Evaluation of a diphenylphosphorylazide-crosslinked collagen membrane for guided bone regeneration in mandibular defects in rats. J. Periodontol..

[27] Behring J., Junker R., Walboomers X.F., Chessnut B., Jansen J.A. (2008). Toward guided tissue and bone regeneration: Morphology, attachment, proliferation, and migration of cells cultured on collagen barrier membranes. A systematic review. Odontology.

[28] Bunyaratavej P., Wang H.L. (2001). Collagen membranes: A review. J. Periodontol..

[29] Moses O., Vitrial D., Aboodi G., Sculean A., Tal H., Kozlovsky A., Artzi Z., Weinreb M., Nemcovsky C.E. (2008). Biodegradation of three different collagen membranes in the rat calvarium: A comparative study. J. Periodontol..

[30] Bornstein M.M., Heynen G., Bosshardt D.D., Buser D. (2009). Effect of two bioabsorbable barrier membranes on bone regeneration of standardized defects in calvarial bone: A comparative histomorphometric study in pigs. J. Periodontol..

[31] Haugh M.G., Jaasma M.J., O’Brien F.J. (2009). The effect of dehydrothermal treatment on the mechanical and structural properties of collagen-GAG scaffolds. J. Biomed. Mater. Res. A.

[32] Torres D.S., Freyman T.M., Yannas I.V., Spector M. (2000). Tendon cell contraction of collagen-GAG matrices in vitro: Effect of cross-linking. Biomaterials.

[33] Moshnikova A.B., Afanasyev V.N., Proussakova O.V., Chernyshov S., Gogvadze V., Beletsky I.P. (2006). Cytotoxic activity of 1-ethyl-3-(3-dimethylaminopropyl)-carbodiimide is underlain by DNA interchain cross-linking. Cell. Mol. Life Sci..

[34] Hafemann B., Ghofrani K., Gattner H.G., Stieve H., Pallua N. (2001). Cross-linking by 1-ethyl-3-(3-dimethylaminopropyl)-carbodiimide (EDC) of a collagen/elastin membrane meant to be used as a dermal substitute: Effects on physical, biochemical and biological features in vitro. J. Mater. Sci. Mater. Med..

[35] Sallent I., Capella-Monsonís H., Zeugolis D.I. (2019). Production and characterization of chemically cross-linked collagen scaffolds. Collagen.

[36] Yamauchi K., Goda T., Takeuchi N., Einaga H., Tanabe T. (2004). Preparation of collagen/calcium phosphate multilayer sheet using enzymatic mineralization. Biomaterials.

[37] Delgado L.M., Bayon Y., Pandit A., Zeugolis D.I. (2015). To cross-link or not to cross-link? Cross-linking associated foreign body response of collagen-based devices. Tissue Eng. Part B.

[38] Rothamel D., Schwarz F., Sager M., Herten M., Sculean A., Becker J. (2005). Biodegradation of differently cross-linked collagen membranes: An experimental study in the rat. Clin. Oral Implant. Res..

[39] Park S.N., Park J.C., Kim H.O., Song M.J., Suh H. (2002). Characterization of porous collagen/hyaluronic acid scaffold modified by 1-ethyl-3-(3-dimethylaminopropyl)carbodiimide cross-linking. Biomaterials.

[40] Campiglio C.E., Contessi Negrini N., Fare S., Draghi L. (2019). Cross-Linking Strategies for Electrospun Gelatin Scaffolds. Materials.

[41] Bae E.B., Kim H.J., Ahn J.J., Bae H.Y., Kim H.J., Huh J.B. (2019). Comparison of Bone Regeneration between Porcine-Derived and Bovine-Derived Xenografts in Rat Calvarial Defects: A Non-Inferiority Study. Materials.

[42] Wang K., Abdala A., Hilal N., Khraisheh M. (2017). Mechanical characterization of membranes. Membrane Characterization.

[43] Schlegel A., Möhler H., Busch F., Mehl A.J.B. (1997). Preclinical and clinical studies of a collagen membrane (Bio-Gide^®^). Biomaterials.

[44] Moses O., Pitaru S., Artzi Z., Nemcovsky C.E. (2005). Healing of dehiscence-type defects in implants placed together with different barrier membranes: A comparative clinical study. Clin. Oral Implant. Res..

[45] Rothamel D., Schwarz F., Sculean A., Herten M., Scherbaum W., Becker J. (2004). Biocompatibility of various collagen membranes in cultures of human PDL fibroblasts and human osteoblast-like cells. Clin. Oral Implant. Res..

[46] Schwarz F., Rothamel D., Herten M., Wüstefeld M., Sager M., Ferrari D., Becker J. (2008). Immunohistochemical characterization of guided bone regeneration at a dehiscence-type defect using different barrier membranes: An experimental study in dogs. Biomaterials.

[47] Powell H.M., Boyce S.T. (2006). EDC cross-linking improves skin substitute strength and stability. Biomaterials.

[48] Angele P., Abke J., Kujat R., Faltermeier H., Schumann D., Nerlich M., Kinner B., Englert C., Ruszczak Z., Mehrl R. (2004). Influence of different collagen species on physico-chemical properties of crosslinked collagen matrices. Biomaterials.

[49] Ghodbane S.A., Dunn M.G. (2016). Physical and mechanical properties of cross-linked type I collagen scaffolds derived from bovine, porcine, and ovine tendons. J. Biomed. Mater. Res. A.

[50] Speer D.P., Chvapil M., Eskelson C.D., Ulreich J. (1980). Biological effects of residual glutaraldehyde in glutaraldehyde-tanned collagen biomaterials. J. Biomed. Mater. Res..

[51] Vizarova K., Bakos D. (1995). Modification of Layered Atelocollagen—Enzymatic Degradation and Cytotoxicity Evaluation. Biomaterials.

[52] Bozkurt S.B., Hakki S.S., Hakki E.E., Durak Y., Kantarci A. (2017). Porphyromonas gingivalis Lipopolysaccharide Induces a Pro-inflammatory Human Gingival Fibroblast Phenotype. Inflammation.

[53] Qiu Y.L., Chen X., Hou Y.L., Hou Y.J., Tian S.B., Chen Y.H., Yu L., Nie M.H., Liu X.Q. (2019). Characterization of different biodegradable scaffolds in tissue engineering. Mol. Med. Rep..

[54] Warnke P.H., Douglas T., Sivananthan S., Wiltfang J., Springer I., Becker S.T. (2009). Tissue engineering of periosteal cell membranes in vitro. Clin. Oral Implant. Res..

[55] Park S.N., Kim J.K., Suh H. (2004). Evaluation of antibiotic-loaded collagen-hyaluronic acid matrix as a skin substitute. Biomaterials.

[56] Li H., Zheng J., Zhang S., Yang C., Kwon Y.D., Kim Y.J. (2018). Experiment of GBR for repair of peri-implant alveolar defects in beagle dogs. Sci. Rep..

[57] Gielkens P.F., Schortinghuis J., de Jong J.R., Raghoebar G.M., Stegenga B., Bos R.R. (2008). Vivosorb^®^, Bio-Gide^®^, and Gore-Tex^®^ as barrier membranes in rat mandibular defects: An evaluation by microradiography and micro-CT. Clin. Oral Implant. Res..

